# Regular Dental Check-Ups Are Associated with Choosing Uninsured Dental Restoration/Prosthesis Treatment in Japan

**DOI:** 10.3390/healthcare11111582

**Published:** 2023-05-28

**Authors:** Katsuo Oshima

**Affiliations:** Department of Dental Technology, The Nippon Dental University College, Tokyo 102-8159, Japan; oshima@tky.ndu.ac.jp

**Keywords:** dental check-ups, uninsured treatment, oral health, health policy, public health insurance system, socioeconomic factors, Japan

## Abstract

Since Japan has implemented Universal Health Coverage (UHC), most dental treatments are covered by public health insurance. Therefore, when receiving fixed dental restoration/prosthesis (FDRP) treatment, such as inlays, crowns, and bridges, the patient can choose whether or not it is covered by insurance. This study aimed to evaluate whether those who receive dental check-ups regularly chose uninsured FDRP treatment. Data were collected from 2088 participants, who had undergone FDRP treatment, via a web-based survey and analyzed. Among them, 1233 (59.1%) had received regular dental check-ups (RDC group) and 855 (40.9%) had not (non-RDC group). The multivariate logistic regression model showed that compared to the non-RDC group, those in the RDC group were statistically significantly associated with higher rates of good oral health behaviors (brushing teeth ≥ 3 times daily, odds ratios (OR):1.46; practiced interdental cleaning habitually, OR: 2.22) and received uninsured FDRP treatment more often (OR: 1.59), adjusted for socioeconomic factors. These results suggest that health policy interventions to promote access to RDC among individuals may improve the oral health of people and reduce the financial burden on the public health insurance system.

## 1. Introduction

Universal Health Coverage (UHC) is a key issue in healthcare policies globally and a target of the Sustainable Development Goals (SDGs) [1]. In many countries, the establishment and continuation of a UHC system are expected to allow more people access to medical and dental care and services, regardless of inequalities due to socioeconomic factors [1,2,3]. However, to maintain a UHC system in each country, it is necessary to secure stable financial resources [4,5]. Therefore, policymakers must plan health policies that reduce excessive increases in healthcare expenses and sustain financial resources. 

Japan has established public health insurance coverage for all its citizens, including medical and dental care [6]. Therefore, the Japanese healthcare system has achieved UHC. Depending on age and income, patients who receive treatment pay between 10% to 30% of out-of-pocket healthcare expenses [6]. The public health insurance system covers most dental care treatments, including endodontic, periodontal, dental restorative, and prosthetic treatments. However, some dental restorative/prosthetic, dental implant, and orthodontic treatments are not. Dental restoration is usually covered by public health insurance. However, if patients require further aesthetic and expensive materials, they can choose uninsured treatment with full out-of-pocket expenses. 

Japanese healthcare spending is increasing yearly due to the aging population and the introduction of new healthcare technologies [7,8]. Although Japan has an insurance-based health coverage system, the tax supplement to healthcare expenditure is extremely high (approximately 40% of the total healthcare expenditure). Hence, there is fear that a further increase in healthcare expenditure will constrain the country’s finances [9]. Regarding dental healthcare expenditure, dental restorative/prosthetic treatment accounts for the highest proportion (32.4%) compared to other dental care, such as basic and administrative expenses (27.1%), dental procedures (23.5%), and others (17.0%) [10]. In addition, the number of patients requiring prosthetic treatment covered by public health insurance is projected to remain constant [11]. 

Health policies to prevent dental diseases and protect oral health are required to address these current conditions. Regular dental utilization for early detection and care is an important health behavior for maintaining oral health [12,13,14]. People who regularly access dental services have a high awareness of oral health, including practicing brushing and daily interdental cleaning of teeth [14,15]. Moreover, previous studies reported that people who regularly had their oral health assessed and strived to prevent dental disease had lower dental healthcare expenses than those who did not [16,17,18]. Therefore, these studies suggest that health policies that guarantee more people access to regular dental check-ups could protect their oral health and reduce excessive increases in healthcare expenditures. In addition, those with an awareness of the importance of oral health are more likely to be willing to pay higher costs for dental care [19,20,21]. Given this finding, those who received regular dental check-ups to protect their oral health may choose uninsured treatments for higher quality materials; however, no such studies have been conducted. 

Hence, the following study was conducted to obtain baseline data for policy planning to reduce excessive increases in public healthcare expenditures. First, study participants who reported receiving treatment for fixed dental restorations/prostheses (FDRP), such as inlays, crowns, and bridges, which are prevalent treatments among Japanese people [22], were sampled from the national population. Then, using participants’ data, an analysis was performed focusing on whether those who regularly received dental check-ups were more likely to choose uninsured FDRP treatment.

## 2. Materials and Methods

### 2.1. Study Setting and Preparing a Dataset of the Participants

This study’s target population was extracted from the registrants of a company that specialized in web research (Macromill Inc., Tokyo, Japan). It had a pool of approximately 1.3 million registered individuals, representing 1% of the Japanese population [23]. 

Participants were recruited through the following process to prepare a dataset for analysis. First, individuals were extracted from the company’s registrants via quota sampling to recruit people nationwide. The extraction criteria were based on the National Census [23] as follows: sex (men and women), age group (20s, 30s, 40s, 50s, and 60s), and regional block (Hokkaido, Tohoku, Kanto, Chubu, Kinki, Chugoku, Shikoku, and Kyushu-region). Japan has a population of approximately 75 million residents aged 20–69 years. Based on an error margin of 2%, 95% confidence coefficient, and 50% population proportion, a minimum sample size of 2401 participants was required. Additionally, a sample size of 3200 participants aged 20–69 years was targeted, considering the number of people who have received FDRP treatment [22]. Consequently, 3336 individuals were sampled. Among them, those who self-reported wearing inlays, crowns, and bridges were selected. They were asked to select one response for each inlay/crown and bridge: “none”, “in upper jaw only”, “in lower jaw only”, or “in both upper and lower jaws”. Those with one or more FDRPs, defined as inlays/crowns or bridges, were selected based on their response. This study focused on inlays, crowns, and bridges because they were considered restorative and prosthetic dental treatments among Japanese people and utilized for various ages [22]. The participants’ self-reported assessment of oral status has been reported in previous studies for validity and usefulness; however, it is imperfectly matched when compared to clinical oral diagnoses by dentists [24,25,26]. Finally, a dataset of 2088 participants, who self-reported wearing FDRP, was prepared. 

A web-based survey was conducted between 12–14 October 2022. Before the survey, the author and staff of the research company evaluated it for ease of use, item consistency, and text correctness. All survey items were in Japanese. The purpose of the study was presented to the study participants, and only those who agreed to participate were included. The participants were required to answer the question on the screen before proceeding to the following question. Thus, there were no missing values. The web research company placed a strict policy to maintain registrants who responded appropriately and excluded those who responded inappropriately [27]. 

### 2.2. Outcome Variable

The outcome variable was whether the participants had received regular dental check-ups. Per a report from the Ministry of Health, Labour and Welfare of Japan, the criterion for regular dental check-ups was defined as [28,29]: “receiving at least one dental check-up during a year.” The participants were divided into two groups: those who underwent regular dental check-ups (RDC group) and those who did not (non-RDC group). 

### 2.3. Explanatory Variables

The explanatory variables were set as the participants’ individual characteristics. They consisted of variables related to “1. individual socioeconomic factors”, “2. oral health status”, and “3. whether or not the FDRP treatment was covered by insurance”. 

1. Individual socioeconomic factors were sex, age, household income, working status, marital status, and the municipality of residence. Age was categorized into five groups: 20–29, 30–39, 40–49, 50–59, or 60–69 years. Household income was categorized into five groups: JPY <4 million, JPY 4–6 million, JPY 6–8 million, JPY ≥8 million, or unknown (as of 2020, the average household income and median were JPY 5.64 and 4.4 million, respectively [30]). Working status was classified into four types: regular workers, homemakers, part-time workers, or unemployed/others. Marital status was categorized into two groups: married or unmarried. Participants’ resident municipalities were differentiated into four groups based on Japan’s municipality system: metropolises (ordinance-designated cities with populations of ≥500,000 and the 23 wards of Tokyo), core cities (≥200,000 population), other cities (≥50,000 population, which excluded metropolises and core cities), or towns and villages (small municipalities that were not qualified as cities). 

2. Oral health status variables were the frequency of tooth brushing and habitual interdental cleaning status. The frequency of tooth brushing was categorized into four groups: ≥3 times, twice, or once a day; or sometimes/no brushing. Habitual interdental cleaning was categorized as yes or no. 

3. Regarding whether FDRP treatment was covered by insurance, the participants were asked if they had ever received one or more uninsured treatments for FDRP. Uninsured FDRP treatment status was categorized as yes or no.

### 2.4. Statistical Analysis

Descriptive statistics were calculated for the participants’ characteristics. All the variables were treated as categorical variables. 

The participants were divided into two groups: RDC and non-RDC. Subsequently, the association between the outcome variables (RDC and non-RDC groups) and explanatory variables (individual socioeconomic factors, oral health status, and uninsured FDRP treatment status) were evaluated via cross-tabulation. The association between the RDC and non-RDC groups and uninsured FDRP treatment is shown in the graph. A chi-squared test was used to compare the proportions of each variable (in addition, a cross-tabulation analysis was conducted on the association between the outcome and explanatory variables, separately by sex, for reference as supplemental data).

A logistic regression analysis was conducted to analyze the individual characteristics of participants in the RDC and non-RDC groups. The outcome variable was whether they had received regular dental check-ups (RDC group = 1, non-RDC group = 0). Furthermore, the explanatory variables comprised individual socioeconomic factors (sex, age, household income, working and marital status, and municipality of residence), oral health status (frequency of tooth brushing and interdental cleaning habits), and uninsured FDRP treatment status. Univariate and multivariate logistic regression analyses via forced entry were conducted to calculate the odds ratios (OR) and 95% confidence intervals (95%CI) for each explanatory variable. The fit of each model was evaluated using significance tests. (Additionally, a logistic regression analysis was conducted on the association between the outcome and explanatory variables, separately by sex, for reference as supplemental data).

Stata version 17 (StataCorp LLC, College Station, TX, USA) was used for data management and all statistical analyses. Statistical significance was set at *p* < 0.05. 

### 2.5. Ethical Considerations

All the participants were individuals registered with a web research company (Macromill, Inc., Tokyo, Japan). They consented to participate and have their data analyzed. Their personal information was protected by the company and could not be identified during the analysis process [31]. The participants were allotted points that could be redeemed for merchandise or converted to cash once they completed the survey. This study was approved by the Research Ethics Committee of the Nippon Dental University College in Tokyo (August 2022, #293). 

## 3. Results

### 3.1. Participants’ Characteristics, the Association of the Proportions between the RDC and Non-RDC Groups, and Their Individual Characteristics

Table 1 presents the participants’ individual characteristics, the association of the proportions between the RDC and non-RDC groups, and their individual characteristics by cross-tabulation. (The results of the association between the RDC and non-RDC groups and their individual characteristics by sex are shown in Supplementary Tables S1 (men) and S2 (women)).

Of the 2088 participants, 1233 (59.1%) and 855 (40.9%) were in the RDC and non-RDC groups, respectively. The chi-squared test revealed statistically significant differences in sex (χ^2^(1) = 26.01, *p* < 0.001), household income (χ^2^(4) = 15.09, *p* = 0.005), municipality of residence (χ^2^(3) = 21.01, *p* < 0.001), frequency of tooth brushing (χ^2^(3) = 38.87, *p* < 0.001), interdental cleaning (χ^2^(1) = 90.04, *p* < 0.001), and uninsured FDRP treatment (χ^2^(1) = 22.22, *p* < 0.001).

**Figure 1:**
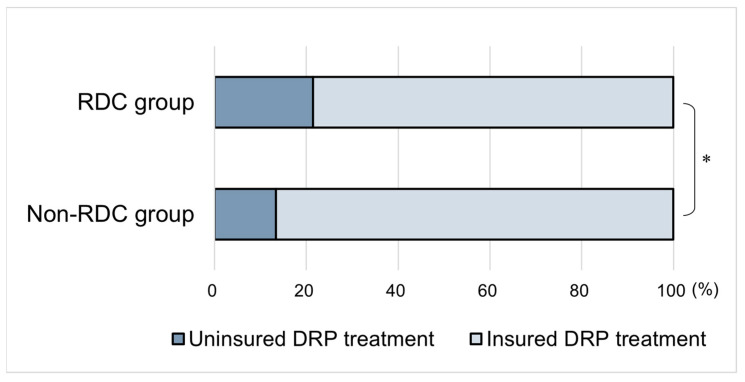
The proportion of uninsured and insured FDRP treatment in the two groups. Note: RDC group = those who received regular dental check-ups; FDRP: fixed dental restorations/prostheses; total number: 2088 participants; of 1233 in the RDC group, 21.4% were uninsured; of the 855 in the non-RDC group, 13.3% were uninsured; * chi-squared test, *p* < 0.001.

Figure 1 illustrates the proportion of those with uninsured FDRP treatment in the RDC (21.4%) and non-RDC groups (13.3%) (*p* < 0.001).

### 3.2. Participants’ Individual Characteristics in the RDC Group Compared to the Non-RDC Group

Table 2 presents the OR and 95%CI in the univariate and multivariate logistic regression analyses for the RDC group compared with the non-RDC group, adjusted for their association with participants’ characteristics (RDC group = 1, non-RDC group = 0). (The results of participants’ individual characteristics in the RDC group compared to the non-RDC group by sex are shown in Supplementary Tables S3 (men) and S4 (women)). 

In the univariate logistic regression analysis, those in the RDC group were statistically significantly lower among men (OR: 0.63, 95%CI: 0.53–0.76), older age groups (40–49 years, OR: 0.60, 95%CI: 0.42–0.85; 50–59 years, OR: 0.64, 95%CI: 0.45–0.91; 60–69 years, OR: 0.67, 95%CI: 0.47–0.95), those unemployed/others (OR: 0.74, 95%CI: 0.57–0.96), and living in rural areas (towns and villages, OR: 0.66, 95%CI: 0.49–0.90). Furthermore, it was higher among those living in urban areas (metropolis, OR: 1.31, 95%CI: 1.07–1.61), had a habit of brushing their teeth (≥3 times daily, OR: 1.81, 95%CI: 1.39–2.36; twice daily, OR: 1.39, 95%CI: 1.10–1.76; sometimes/no brushing, OR: 0.28, 95%CI: 0.12–0.63), had interdental cleaning habits (OR: 2.43, 95%CI: 2.02–2.92), and who received uninsured FDRP treatment (OR: 1.77, 95%CI: 1.39–2.25). 

In the multivariate logistic regression analysis, those in the RDC group were statistically significantly lower among men (OR: 0.66, 95%CI: 0.52–0.84), older age groups (30–39 years, OR: 0.64, 95%CI: 0.42–0.96; 40–49 years, OR: 0.48, 95%CI: 0.33–0.70; 50–59 years, OR: 0.54, 95%CI: 0.37–0.79; 60–69 years, OR: 0.53, 95%CI: 0.36–0.79), homemakers (OR: 0.63, 95%CI: 0.46–0.87), and those who lived in rural areas (towns and villages, OR: 0.66, 95%CI: 0.48–0.90). Furthermore, it was higher in those were married (OR: 1.33, 95%CI: 1.06–1.67), had a habit of brushing their teeth (≥3 times daily, OR: 1.46, 95%CI: 1.10–1.94; sometimes/no brushing, OR: 0.39, 95%CI: 0.17–0.89), had interdental cleaning habits (OR: 2.22, 95%CI: 1.83–2.70), and received uninsured FDRP treatment (OR: 1.59, 95%CI: 1.23–2.04). 

## 4. Discussion

### 4.1. Key Findings

This study used a web-based survey and extracted data from those who received FDRP treatment among a Japanese nationwide scale population to create a dataset. Furthermore, the participants’ individual characteristics between the RDC and non-RDC groups were analyzed. Of the 2088 participants, 1233 (59.1%) were in the RDC group, and 855 (40.9%) were in the non-RDC group. The multivariate logistic regression analysis revealed that compared to the non-RDC group, those in the RDC group were significantly higher in those who brushed their teeth, had interdental cleaning habits, and received uninsured FDRP treatment, adjusted for socioeconomic factors. 

These results suggested that those who received regular dental check-ups practiced good oral health behaviors and could be inclined to choose uninsured FDRP treatment. 

### 4.2. Association between the RDC/Non-RDC Groups and Their Individual Characteristics

Maintaining good oral health requires the regular utilization of dental services and self-protection, such as oral hygiene, fluoride usage, and moderate sugar consumption. Regarding oral hygiene, tooth brushing, and habitual interdental cleaning were important strategies to prevent dental problems and periodontal disease [32,33]. Furthermore, those with regular dental attendance were more likely to practice appropriate oral health behaviors daily, such as tooth brushing and interdental cleaning [14,15]. Regarding oral health behaviors (Table 2), the RDC group was more inclined to practice habitual tooth brushing and interdental cleaning than the non-RDC group, which supported the results of previous studies [14,15]. 

Regarding the other primary result, the RDC group was statistically significantly associated with choosing uninsured FDRP treatment compared with the non-RDC group. A Swedish study [25] demonstrated that the group that self-reported using fixed restorations, such as crowns, bridges, and implants, was willing to pay more for oral care. This result suggested that the group that used fixed restorations found it important enough to prioritize their oral health. In addition, previous studies reported that those with a higher awareness of oral health were willing to pay higher dental care costs, including implants [19,20] and filling [21]. Thus, consistent with previous research findings, this study’s results suggested that those with high awareness of oral health who practiced RDC were more likely to pay willingly higher expenses for FDRP treatment, which included choosing uninsured treatments with aesthetic and expensive materials. 

In contrast, regarding the association between those who received RDC and household income, the analysis of only women (Supplementary file, Table S4) indicated a slight association with those who received more than JPY 8 million. However, an analysis of the total participants showed no statistically significant association. Several previous studies reported an association between regular dental attendance and a higher income [13,14,34,35]; however, these reports differed from the results of this study. This may be because the study’s dataset was based on individuals who self-reported receiving dental restorative/prosthetic treatment. A previous nationwide study with the general population sampled from registrants of the same web research company showed a statistically significant association between receiving RDC and higher incomes [14].

Although the results of this study revealed no statistically significant association between the RDC group and household income, the following results may suggest an association between the RDC group and economic status. First, the RDC group had more married individuals and fewer homemakers than the non-RDC group. A possible reason for this result was that paying for dental check-ups was a lower priority for homemakers than household expenditures. In addition, the RDC group has fewer individuals in rural areas, possibly due to differences in economic status between urban and rural areas, [30] rather than the geographic maldistribution of dental services [36]. However, the association between the choice of uninsured treatment and an individual’s economic status was difficult to explain based on these results. Hence, the results should be verified separately.

### 4.3. Implications for Health Policy

A focal result of this study was that the RDC group was inclined to choose uninsured FDRP treatment compared to the non-RDC group in Japan, a country with a public health insurance system. This suggested that those with a higher awareness of protecting oral health were willing to pay higher costs for dental treatment. Furthermore, previous studies reported that those who protected their oral health through regular dental attendance were more likely to have lower dental healthcare expenses [16,17,18]. Therefore, considered in conjunction with this study’s results, promoting the habit of receiving RDC may lead to a reduced burden on public insurance funding. 

To permanently maintain a UHC system, it is necessary to pool financial resources [4,5]. Japan has UHC and a generous policy regarding its dental care system, as restorative/prosthetic treatments are covered by public insurance [10]. However, there has been an impact on public healthcare funding [7]. Therefore, these findings could provide data that could serve as evidence for health policies that promote more people to receive RDC from the perspective of optimizing healthcare costs to sustain UHC. The public health insurance system is a meaningful policy for maintaining oral health status, even for the economically poor. Simultaneously, the choice of uninsured treatment by the economically affluent may reduce pressure on resources. In addition to securing funding to sustain UHC, policymakers should accumulate further evidence to curb healthcare costs by promoting oral health. 

### 4.4. Limitations

This study has several limitations. First, it assessed the status of FDRP treatment based on participants’ self-report using a web-based survey, which was not ascertained through a clinical examination by a dentist. However, previous studies confirmed the validity of self-report assessments by the general population regarding oral conditions, such as dental restorative prosthetics [24,25,26]. In addition, the web research company placed a strict policy to maintain registrants who responded appropriately by deleting the registrations of those who responded inappropriately. However, it was unclear how consistent the participants’ self-report of their oral status was with their actual oral status. Second, at the initial stage of recruiting participants, this study was set up for distribution according to sex, age group, and regional block using a quota sampling method to approximate the distribution of the Japanese population nationwide. However, the composition of the participants within each group (sex, age group, and regional block) did not reflect the entire Japanese population. Therefore, sampling bias may have occurred among the participants. Third, this study primarily revealed an association between those who received RDC and the status of uninsured FDRP treatment. However, since it was a cross-sectional design, it was impossible to assess causal relationships between each factor. Further research is required to determine the impact of habitual RDC visits on the choice of uninsured treatments. 

## 5. Conclusions

This study used a web-based survey targeting those who had undergone FDRP treatment and analyzed participants’ individual characteristics between the RDC and non-RDC groups. The results revealed that compared to the non-RDC group, the RDC group was more likely to practice good oral health behaviors (brushing teeth ≥ 3 times daily, OR:1.46; practiced interdental cleaning habitually, OR: 2.22) and choose uninsured FDRP treatment (OR: 1.59).

These results suggest that health policy interventions to promote regular access to dental check-ups among the general population may contribute to improving their oral health and reducing the financial burden on the public health insurance system.

## Figures and Tables

**Table 1:** Participants’ characteristics, the association between the RDC and the non-RDC groups, and their individual characteristics.

	Total Number	Whether or not the Participants Received RDC					
		RDC Group		Non-RDC Group		χ^2^-Value	*p*-Value
Total	2088	1233	(59.1)	855	(40.9)		
Sex, *n* (%)							
Men	974	518	(53.2)	456	(46.8)	χ^2^(1) = 26.01	<0.001
Women	1114	715	(64.2)	399	(35.8)		
Age, *n* (%)							
20–29 years	183	125	(68.3)	58	(31.7)	χ^2^(4) = 8.68	0.070
30–39 years	321	194	(60.4)	127	(39.6)		
40–49 years	508	286	(56.3)	222	(43.7)		
50–59 years	533	308	(57.8)	225	(42.2)		
60–69 years	543	320	(58.9)	223	(41.1)		
Household income, *n* (%)							
JPY <4 million	549	320	(58.3)	229	(41.7)	χ^2^(4) = 15.09	0.005
JPY 4–6 million	419	255	(60.9)	164	(39.1)		
JPY 6–8 million	314	194	(61.8)	120	(38.2)		
JPY ≥8 million	379	243	(64.1)	136	(35.9)		
Unknown	427	221	(51.8)	206	(48.2)		
Working status, *n* (%)							
Regular worker	1105	663	(60.0)	442	(40.0)	χ^2^(3) = 5.67	0.129
Homemaker	385	231	(60.0)	154	(40.0)		
Part-time worker	320	193	(60.3)	127	(39.7)		
Unemployed/others	278	146	(52.5)	132	(47.5)		
Marital status, *n* (%)							
Married	1324	802	(60.6)	522	(39.4)	χ^2^(1) = 3.47	0.063
Unmarried	764	431	(56.4)	333	(43.6)		
Municipalities, *n* (%)							
Metropolis (pop 500,000+)	736	468	(63.6)	268	(36.4)	χ^2^(3) = 21.01	<0.001
Core cities (pop 200,000+)	343	210	(61.2)	133	(38.8)		
Cities (pop 50,000+)	796	455	(57.2)	341	(42.8)		
Towns and villages	213	100	(46.9)	113	(53.1)		
Frequency of brushing teeth, *n* (%)							
≥Three times daily	576	378	(65.6)	198	(34.4)	χ^2^(3) = 38.87	<0.001
Twice daily	1093	650	(59.5)	443	(40.5)		
Once daily	384	197	(51.3)	187	(48.7)		
Sometimes/No brushing	35	8	(22.9)	27	(77.1)		
Interdental cleaning, *n* (%)							
Yes	862	614	(71.2)	248	(28.8)	χ^2^(1) = 90.04	<0.001
No	1226	619	(50.5)	607	(49.5)		
Uninsured FDRP treatment, *n* (%)							
Yes	378	264	(69.8)	114	(30.2)	χ^2^(1) = 22.22	<0.001
No	1710	969	(56.7)	741	(43.3)		

Note: RDC group = group of those who received regular dental check-ups; χ^2^-chi-squared test.

**Table 2:** Participants’ characteristics in the RDC group compared to the non-RDC group (logistic regression analysis, RDC group = 1; non-RDC group = 0).

	Univariate Analysis			Multivariate Adjustment Model		
	OR	95%CI	*p*-Value	OR	95%CI	*p*-Value
Sex						
Men	0.63	(0.53–0.76)	<0.001	0.66	(0.52–0.84)	0.001
Women	Reference			Reference		
Age						
20–29 years	Reference			Reference		
30–39 years	0.71	(0.48–1.04)	0.078	0.64	(0.42–0.96)	0.030
40–49 years	0.60	(0.42–0.85)	0.005	0.48	(0.33–0.70)	<0.001
50–59 years	0.64	(0.45–0.91)	0.012	0.54	(0.37–0.79)	0.001
60–69 years	0.67	(0.47–0.95)	0.025	0.53	(0.36–0.79)	0.002
Household income						
JPY <4 million	Reference			Reference		
JPY 4–6 million	1.11	(0.86–1.44)	0.420	0.98	(0.74–1.30)	0.881
JPY 6–8 million	1.16	(0.87–1.54)	0.314	1.06	(0.77–1.45)	0.738
JPY ≥8 million	1.28	(0.98–1.67)	0.074	1.04	(0.76–1.42)	0.802
Unknown	0.77	(0.60–0.99)	0.042	0.75	(0.57–0.98)	0.036
Working status						
Regular worker	Reference			Reference		
Homemaker	1.00	(0.79–1.27)	1.000	0.63	(0.46–0.87)	0.004
Part-time worker	1.01	(0.79–1.31)	0.920	0.81	(0.60–1.10)	0.178
Unemployed/others	0.74	(0.57–0.96)	0.024	0.79	(0.58–1.07)	0.131
Marital status						
Married	1.19	(0.99–1.42)	0.063	1.33	(1.06–1.67)	0.016
Unmarried	Reference			Reference		
Municipalities						
Metropolis (pop 500,000+)	1.31	(1.07–1.61)	0.010	1.22	(0.98–1.52)	0.072
Core cities (pop 200,000+)	1.18	(0.91–1.53)	0.202	1.19	(0.91–1.56)	0.211
Cities (pop 50,000+)	Reference			Reference		
Towns and villages	0.66	(0.49–0.90)	0.008	0.66	(0.48–0.90)	0.009
Frequency of brushing teeth						
≥Three times daily	1.81	(1.39–2.36)	<0.001	1.46	(1.10–1.94)	0.008
Twice daily	1.39	(1.10–1.76)	0.005	1.16	(0.90–1.49)	0.243
Once daily	Reference			Reference		
Sometimes/No brushing	0.28	(0.12–0.63)	0.002	0.39	(0.17–0.89)	0.025
Interdental cleaning						
Yes	2.43	(2.02–2.92)	<0.001	2.22	(1.83–2.70)	<0.001
No	Reference			Reference		
Uninsured FDRP treatment						
Yes	1.77	(1.39–2.25)	<0.001	1.59	(1.23–2.04)	<0.001
No	Reference			Reference		

Note: RDC group = those who received regular dental check-ups; OR = odds ratio; 95%CI = 95% confidence interval; multivariate adjustment model: number of observations = 2088, χ^2^(21) = 193.36; log-likelihood = −1316.21; *p* < 0.001.

## Data Availability

Data cannot be shared publicly as no informed consent was provided by the participants for open data sharing.

## Supplementary Materials

The following supporting information can be downloaded at: https://www.mdpi.com/article/10.3390/healthcare11111582/s1, Table S1: Results of the association between the RDC and non-RDC groups and their individual characteristics by sex (men); Table S2 Results of the association between the RDC and non-RDC groups and their individual characteristics by sex (women); Table S3: Results of the participants’ individual characteristics in the RDC group compared to the non-RDC group by sex (men); Table S4: Results of the participants’ individual characteristics in the RDC group compared to the non-RDC group by sex (women).
