# Supplementary material for: Regular Dental Check-Ups Are Associated with Choosing Uninsured Dental Restoration/Prosthesis Treatment in Japan
Source: Healthcare (Basel). 2023 May 28;11(11):1582. doi: 10.3390/healthcare11111582 (PMC10252183; doi:10.3390/healthcare11111582)
Supplement: Supplementary file 1 [file healthcare-11-01582-s001.zip › healthcare-2395910-supplementary.pdf]

Supplementary file: Table S1

Table S1. Male participants' characteristics, the association between the RDC and the non-RDC groups, and their individual characteristics.

	Total Number	Whether or not the participants receive RDC					
		RDC group		non-RDC group		χ^2-Value	p-Value
Total	974	518	(53.2)	456	(46.8)		
Age, n (%)							
20–29 years	73	44	(60.3)	29	(39.7)	$\chi^2(4)$ = 2.47	0.651
30–39 years	159	84	(52.8)	75	(47.2)		
40–49 years	222	111	(50.0)	111	(50.0)		
50–59 years	261	139	(53.3)	122	(46.7)		
60–69 years	259	140	(54.1)	119	(45.9)		
Household income, n (%)							
<4 million JPY	213	110	(51.6)	103	(48.4)	$\chi^2(4)$ = 7.60	0.107
4–6 million JPY	205	108	(52.7)	97	(47.3)		
6–8 million JPY	164	94	(57.3)	70	(42.7)		
≥ 8 million JPY	232	134	(57.8)	98	(42.2)		
Unknown	160	72	(45.0)	88	(55.0)		
Working status, n (%)							
Regular worker	751	412	(54.9)	339	(45.1)	$\chi^2(3)$ = 4.77	0.189
Homemaker	10	6	(60.0)	4	(40.0)		
Part-time worker	47	24	(51.1)	23	(48.9)		
Not working and others	166	76	(45.8)	90	(54.2)		
Marital status, n (%)							
Married	608	350	(57.6)	258	(42.4)	$\chi^2(1)$ = 12.48	<0.001
Single	366	168	(45.9)	198	(54.1)		
Municipalities, n (%)							
Metropolis (pop 500,000+)	323	186	(57.6)	137	(42.4)	$\chi^2(3)$ = 7.67	0.053
Core cities (pop 200,000+)	182	101	(55.5)	81	(44.5)		
Cities (pop 50,000+)	378	192	(50.8)	186	(49.2)		
Towns and villages	91	39	(42.9)	52	(57.1)		
Frequency of brushing teeth, n (%)							
≥ three times daily	215	125	(58.1)	90	(41.9)	$\chi^2(3)$ = 18.42	<0.001
Twice daily	476	268	(56.3)	208	(43.7)		
Once daily	254	118	(46.5)	136	(53.5)		
Sometimes/No brushing	29	7	(24.1)	22	(75.9)		
Interdental cleaning, n (%)							
Yes	289	185	(64.0)	104	(36.0)	$\chi^2(1)$ = 19.36	<0.001
No	685	333	(48.6)	352	(51.4)		
Uninsured FDRP treatment, n (%)							
Yes	137	91	(66.4)	46	(33.6)	$\chi^2(1)$ = 11.23	0.001
No	837	427	(51.0)	410	(49.0)		

Note: RDC group = group of those who received regular dental check-ups; χ^2- chi-squared test.

Supplementary file: Table S2

Table S2. Female participants' characteristics, the association between the RDC and the non-RDC groups, and their individual characteristics.

	Total Number	Whether or not the participants receive RDC					
		RDC group		non-RDC group		χ^2-Value	p-Value
Total	1114	715	(64.2)	399	(35.8)		
Age, n (%)							
20–29 years	110	81	(73.6)	29	(26.4)	$\chi^2(4)$ = 6.94	0.139
30–39 years	162	110	(67.9)	52	(32.1)		
40–49 years	286	175	(61.2)	111	(38.8)		
50–59 years	272	169	(62.1)	103	(37.9)		
60–69 years	284	180	(63.4)	104	(36.6)		
Household income, n (%)							
<4 million JPY	336	210	(62.5)	126	(37.5)	$\chi^2(4)$ = 17.21	0.002
4–6 million JPY	214	147	(68.7)	67	(31.3)		
6–8 million JPY	150	100	(66.7)	50	(33.3)		
≥ 8 million JPY	147	109	(74.1)	38	(25.9)		
Unknown	267	149	(55.8)	118	(44.2)		
Working status, n (%)							
Regular worker	354	251	(70.9)	103	(29.1)	$\chi^2(3)$ = 10.56	0.014
Homemaker	375	225	(60.0)	150	(40.0)		
Part-time worker	273	169	(61.9)	104	(38.1)		
Not working and others	112	70	(62.5)	42	(37.5)		
Marital status, n (%)							
Married	716	452	(63.1)	264	(36.9)	$\chi^2(1)$ = 0.97	0.325
Single	398	263	(66.1)	135	(33.9)		
Municipalities, n (%)							
Metropolis (pop 500,000+)	413	282	(68.3)	131	(31.7)	$\chi^2(3)$ = 14.85	0.002
Core cities (pop 200,000+)	161	109	(67.7)	52	(32.3)		
Cities (pop 50,000+)	418	263	(62.9)	155	(37.1)		
Towns and villages	122	61	(50.0)	61	(50.0)		
Frequency of brushing teeth, n (%)							
≥ three times daily	361	253	(70.1)	108	(29.9)	$\chi^2(3)$ = 13.40	0.004
Twice daily	617	382	(61.9)	235	(38.1)		
Once daily	130	79	(60.8)	51	(39.2)		
Sometimes/No brushing	6	1	(16.7)	5	(83.3)		
Interdental cleaning, n (%)							
Yes	573	429	(74.9)	144	(25.1)	$\chi^2(1)$ = 58.61	<0.001
No	541	286	(52.9)	255	(47.1)		
Uninsured FDRP treatment, n (%)							
Yes	241	173	(71.8)	68	(28.2)	$\chi^2(1)$ = 7.73	0.005
No	873	542	(62.1)	331	(37.9)		

Note: RDC group = group of those who received regular dental check-ups; χ^2- chi-squared test.

Supplementary file: Table S3

Table S3. Male participants' characteristics in the RDC group compared to the non-RDC group (logistic regression analysis, RDC group=1; non-RDC group=0).

	Univariate analysis			Multivariate adjustment model		
	**OR**	**95%CI**	**p-Value**	**OR**	**95%CI**	**p-Value**
Age						
20–29 years	Reference			Reference		
30–39 years	0.74	(0.42-1.30)	0.290	0.64	(0.35-1.16)	0.141
40–49 years	0.66	(0.39-1.13)	0.129	0.51	(0.29-0.90)	0.021
50–59 years	0.75	(0.44-1.27)	0.288	0.61	(0.35-1.07)	0.087
60–69 years	0.78	(0.46-1.32)	0.346	0.56	(0.31-1.00)	0.049
Household income						
<4 million JPY	Reference			Reference		
4–6 million JPY	1.04	(0.71-1.53)	0.832	0.83	(0.54-1.27)	0.387
6–8 million JPY	1.26	(0.83-1.89)	0.273	0.97	(0.60-1.55)	0.886
≥ 8 million JPY	1.28	(0.88-1.86)	0.196	0.82	(0.52-1.27)	0.366
Unknown	0.77	(0.51-1.16)	0.204	0.69	(0.45-1.07)	0.100
Working status						
Regular worker	Reference			Reference		
Homemaker	1.23	(0.35-4.41)	0.746	1.77	(0.44-7.08)	0.420
Part-time worker	0.86	(0.48-1.55)	0.612	1.00	(0.53-1.89)	0.993
Not working and others	0.69	(0.50-0.97)	0.034	0.78	(0.52-1.18)	0.243
Marital status						
Married	1.60	(1.23-2.08)	<0.001	1.57	(1.15-2.13)	0.004
Single	Reference			Reference		
Municipalities						
Metropolis (pop 500,000+)	1.32	(0.98-1.77)	0.072	1.22	(0.89-1.66)	0.221
Core cities (pop 200,000+)	1.21	(0.85-1.72)	0.297	1.18	(0.81-1.71)	0.386
Cities (pop 50,000+)	Reference			Reference		
Towns and villages	0.73	(0.46-1.15)	0.175	0.66	(0.41-1.08)	0.096
Frequency of brushing teeth						
≥ three times daily	1.60	(1.11-2.31)	0.012	1.51	(1.03-2.21)	0.035
Twice daily	1.49	(1.09-2.02)	0.011	1.33	(0.97-1.82)	0.080
Once daily	Reference			Reference		
Sometimes/No brushing	0.37	(0.15-0.89)	0.026	0.43	(0.17-1.09)	0.075
Interdental cleaning						
Yes	1.88	(1.42-2.50)	<0.001	1.82	(1.35-2.44)	<0.001
No	Reference			Reference		
Uninsured FDRP treatment						
Yes	1.90	(1.30-2.78)	0.001	1.90	(1.27-2.84)	0.002
No	Reference			Reference		

Note: RDC group = a group of those who received regular dental check-ups; OR = odds ratio; 95%CI = 95% confidence interval. Multivariate adjustment model: Number of observations = 974, $\chi^2(20)$ = 71.63, Log likelihood = −637.33, $p < 0.001$.

Supplementary file: Table S4

Table S4. Female participants' characteristics in the RDC group compared to the non-RDC group (logistic regression analysis, RDC group=1; non-RDC group=0).

	**Univariate analysis**			**Multivariate adjustment model**		
	**OR**	**95%CI**	**p-Value**	**OR**	**95%CI**	**p-Value**
Age						
20–29 years	Reference			Reference		
30–39 years	0.76	(0.44-1.30)	0.311	0.66	(0.37-1.18)	0.162
40–49 years	0.56	(0.35-0.92)	0.021	0.47	(0.28-0.79)	0.005
50–59 years	0.59	(0.36-0.96)	0.033	0.50	(0.29-0.84)	0.009
60–69 years	0.62	(0.38-1.01)	0.055	0.52	(0.31-0.90)	0.018
Household income						
<4 million JPY	Reference			Reference		
4–6 million JPY	1.32	(0.92-1.89)	0.138	1.15	(0.77-1.71)	0.500
6–8 million JPY	1.20	(0.80-1.80)	0.378	1.16	(0.74-1.82)	0.511
≥ 8 million JPY	1.72	(1.12-2.65)	0.013	1.52	(0.94-2.45)	0.089
Unknown	0.76	(0.55-1.05)	0.096	0.80	(0.56-1.14)	0.220
Working status						
Regular worker	Reference			Reference		
Homemaker	0.62	(0.45-0.84)	0.002	0.66	(0.45-0.98)	0.038
Part-time worker	0.67	(0.48-0.93)	0.018	0.81	(0.55-1.17)	0.258
Not working and others	0.68	(0.44-1.07)	0.095	0.79	(0.49-1.29)	0.348
Marital status						
Married	0.88	(0.68-1.14)	0.325	1.09	(0.76-1.58)	0.629
Single	Reference			Reference		
Municipalities						
Metropolis (pop 500,000+)	1.27	(0.95-1.69)	0.104	1.20	(0.88-1.62)	0.251
Core cities (pop 200,000+)	1.24	(0.84-1.82)	0.282	1.17	(0.78-1.75)	0.450
Cities (pop 50,000+)	Reference			Reference		
Towns and villages	0.59	(0.39-0.89)	0.011	0.66	(0.43-1.01)	0.054
Frequency of brushing teeth						
≥ three times daily	1.51	(1.00-2.30)	0.052	1.27	(0.81-1.99)	0.289
Twice daily	1.05	(0.71-1.55)	0.807	0.94	(0.62-1.42)	0.767
Once daily	Reference			Reference		
Sometimes/No brushing	0.13	(0.01-1.14)	0.065	0.14	(0.02-1.33)	0.087
Interdental cleaning						
Yes	2.66	(2.06-3.42)	<0.001	2.61	(2.00-3.40)	<0.001
No	Reference			Reference		
Uninsured FDRP treatment						
Yes	1.55	(1.14-2.12)	0.006	1.37	(0.98-1.91)	0.065
No	Reference			Reference		

Note: RDC group = a group of those who received regular dental check-ups; OR = odds ratio; 95%CI = 95% confidence interval. Multivariate adjustment model: Number of observations = 1114, $\chi^2(20)$ = 111.89, Log likelihood = −670.78, $p < 0.001$.
